# Targeting the BlyS/APRIL axis in neuro-Behçet disease: a rationale for telitacicept as a steroid-sparing strategy

**DOI:** 10.1097/MS9.0000000000004928

**Published:** 2026-04-17

**Authors:** Muhammad Qaseem, Minhaj Ul Hassan, Aneeq Mahmood Khan, Ayesha Mustafa, Mahnoor Fatima

**Affiliations:** a Department of Medicine, Khyber Medical College, Peshawar, Pakistan; b Department of Medicine, Liaquat University of Medical and Health Sciences (LUMHS), Jamshoro, Pakistan; c Department of Medicine, Sargodha Medical College, Sargodha, Pakistan; d Department of Medicine, Fatima Jinnah Medical University, Pakistan; e Department of Medicine, Shaheed Ziaur Rahman Medical College, Rajshahi Medical University, Bogura, Bangladesh

*To the Editor,*

Neuro-Behçet disease (NBD) is a severe nervous system complication associated with Behçet disease, and its pathogenesis involves immune-mediated inflammation of the central nervous system’s blood vessels, primarily driven by cytokines, including TNF-alpha. This condition arises through clinical manifestations, which include brain stem syndrome, meningoencephalitis, and cerebral venous thrombosis. It is a major contributor to disability and mortality rates, yet it remains underreported in health estimations across the world. Existing medications include corticosteroids and conventional immunosuppressives; on the other hand, a newly identified target therapy called Telitacicept has been developed. Other immune-modulating therapies include infliximab, azathioprine, and interferon alpha^[^1^]^.

Telitacicept consists of a recombinant fusion protein that combines the TACI component with the Fc component, which inhibits the actions of BlyS and APRIL, causing a subsequent reduction in the activation of B-cells and the production of autoantibodies. This provides a scientifically rational approach regarding the drug’s application in the treatment of refractory neuro-Behcet’s disease. In a randomized, double-blind, phase 2b clinical trial among patients with systemic lupus erythematosus (SLE), 75.8% of the subjects receiving the drug Telitacicept demonstrated an SRI-4 response in comparison to 33.9% treated with the placebo. The drug was generally well tolerated, having also been trialed for application in lupus nephritis, myasthenia gravis, and IgA nephropathy^[^2^]^.

The prolonged use of corticosteroids for the management of neuro-Bechet’s disease results in significant adverse effects, and there is a lack of good alternatives to steroids. The immune system of Neuro-Behçet disease (NBD) patients can be effectively modulated through Telitacicept which creates an acceptable path for this purpose. Recent research shows that B-cell activation, together with immune system dysfunctions through humoral mechanisms, operates as a major cause of NBD despite the fact that this disorder does not qualify as an antibody-mediated disease like traditional autoantibody-based illnesses. The research establishes direct mechanistic evidence that B cells participate in neuroinflammation by their presence in the cerebrospinal fluid of NBD patients with elevated BAFF (BLyS) levels, especially in chronic progressive NBD cases^[^3,4^]^. A phase 2 clinical trial study of Telitacicept in adult patients with generalized myasthenia gravis provides translational evidence, in which the primary efficacy endpoint was the mean change in the quantitative myasthenia gravis (QMG) score from baseline. The treatment of roughly 60 patients with Telitacicept 160 mg and 240 mg groups, at week 12, shows mean reductions in QMG scores for these two groups were 5.8 (±5.85) and 9.5 (±5.03), respectively, indicating rapid clinical improvement. Serum IgA, IgG, and IgM levels were consistently reduced by one-third in all patients when compared to placebo^[^5^]^. Similar findings have been reported in

SLE phase 2b double-blinded randomized trial showed significantly higher Systemic Lupus Erythematosus Index 4 (SRI-4) response rate at week 48 (approximately 70–80%) compared to placebo (30–40%). Importantly, patients on high-dose glucocorticoids sustain a reduction in their dosage with Telitacicept use^[^2^]^. When viewed together, these data provide supportive, although indirect evidence that targeting BlyS and APRIL could offer a rational steroid-sparing strategy in NBD, a concept that requires confirmation in prospective studies.

The translational rationale for targeting BlyS and APRIL in NBD is compelling; there are several knowledge gaps and practical challenges remaining. Currently, there is no disease-specific clinical trial evaluating Telitacicept or related agents in NBD. Evidence from other autoimmune diseases cannot be directly generalized, and it should be viewed with appropriate caution. Moreover, experience with long-term inhibition of BlyS/APRIL in neuroinflammatory disorders remains limited, with potential risks including hypogammaglobinemia and infections. Beyond biological uncertainty, real-world barriers such as high treatment cost and limited availability in resource-limited regions restrict the use of Telitacicept^[^6^]^.

The combination of emerging mechanistic insights (including elevated CSF BAFF in NBD) and clinical evidence from related autoimmune diseases shows that existing treatments for neuro-Behçet disease need better options that do not depend on long-term use of corticosteroids^[^1,3^]^. The research community must establish NBD-specific clinical trials that test BlyS/APRIL inhibitors like Telitacicept while developing consistent reporting systems and outcome assessment tools that apply to NBD patients from different demographic backgrounds, testing long-term safety risks in neuroinflammatory conditions and evaluating both steroid-free and steroid-sparing treatment methods and finding solutions for biologic drug access through teamwork between healthcare sectors and advocacy for health policy change^[^2,6^]^.

In conclusion, Telitacicept could represent a potentially steroid-sparing new approach in the management of NBD, based upon the encouraging data obtained in other autoimmune diseases. Nevertheless, its current role cannot be delineated without the conduct of disease-specific clinical trials. Therefore, prospective clinical trials are urgently needed to investigate the potential impact of the modulation of the BlyS/APRIL pathway to reduce steroid-dependence in NBD patients.

This letter is in line with the TITAN Guidelines on the need for transparency in AI use in healthcare^[^7^]^.

## Data Availability

Not applicable to this study.
